# Elevated cerebrospinal fluid protein levels associated with poor short-term outcomes after spinal cord stimulation in patients with disorders of consciousness

**DOI:** 10.3389/fnagi.2022.1032740

**Published:** 2022-11-03

**Authors:** Qiheng He, Tianfei Li, Ying Xiong, Xiaoyu Xia, Yuanyuan Dang, Xueling Chen, Xiaoli Geng, Jianghong He, Yi Yang, Jizong Zhao

**Affiliations:** ^1^Department of Neurosurgery, Beijing Tiantan Hospital, Capital Medical University, Beijing, China; ^2^Department of Neurosurgery, China National Clinical Research Center for Neurological Diseases, Beijing, China; ^3^Department of Neurosurgery, Bozhou Hospital of Traditional Chinese Medicine, Bozhou, China; ^4^Department of Neurosurgery, PLA General Hospital, Beijing, China; ^5^Joint Lab, Chinese Institute for Brain Research, Beijing, China; ^6^Center of Stroke, Beijing Institute of Brain Disorders, Beijing, China

**Keywords:** disorders of consciousness, spinal cord stimulation, cerebrospinal fluid protein, sagittal diameter, outcome

## Abstract

**Background:**

Spinal cord stimulation (SCS) is a promising treatment for patients with disorders of consciousness (DoC); however, the laboratory examinations and different electrodes (permanent #39286 vs. temporary percutaneous #3777, Medtronic, USA) that are associated with postoperative outcomes are unclear. The study aims to study the association between the change in postoperative cerebrospinal fluid (CSF) protein level and improvement in consciousness after SCS in DoC patients and to explore whether different electrodes were associated with elevated CSF protein levels.

**Materials and methods:**

A total of 66 DoC patients who received SCS treatment from December 2019 to December 2021 were retrospectively analyzed. Patients were grouped according to their elevated CSF protein level. The clinical characteristics of the patients and SCS stimulation parameters were compared. The preoperative sagittal diameter of the spinal canal is the distance from the midpoint of the posterior border of the vertebral body to the midpoint of the posterior wall of the spinal canal at the level of the superior border of C3. The postoperative sagittal diameter of the spinal canal is the distance from the midpoint of the posterior edge of the vertebral body to the anterior edge of the stimulation electrode. Patients with improved postoperative CRS-R scores greater than 3 or who progressed to the MCS + /eMCS were classified as the improved group and otherwise regarded as poor outcome.

**Results:**

We found that more DoC patients had elevated CSF protein levels among those receiving SCS treatment with permanent electrodes than temporary percutaneous electrodes (*P* = 0.001), and elevated CSF protein levels were significantly associated with a reduced sagittal diameter (*P* = 0.044). In DoC patients receiving SCS treatment, we found that elevated CSF protein levels (*P* = 0.022) and preoperative diagnosis (*P* = 0.003) were significantly associated with poor outcomes at 3 months. Logistic regression analysis showed that elevated CSF protein levels were significantly associated with poor outcomes (OR 1.008, 95% CI 1.001–1.016, *P* = 0.032).

**Conclusion:**

The results suggest that reducing the effect of electrode pads on anatomical changes may help improve the outcomes of DoC patients receiving SCS treatment. CSF protein levels are associated with poor postoperative outcomes and whether they are potential biomarkers in DoC patients receiving SCS treatment remain further exploration.

## Introduction

Disorders of consciousness (DoC) are among the most frequent complications in patients after severe brain injury and are mainly caused by trauma, stroke, or anoxia (Edlow et al., 2021). DoCs include coma, vegetative state/unresponsive wakefulness syndrome (VS/UWS), and the minimally conscious state (MCS). In recent years, with the development of electrical stimulation technology, neuromodulation technologies represented by deep brain stimulation (DBS) and spinal cord stimulation (SCS) have been increasingly used to treat DoC and have shown encouraging results (Vanhoecke and Hariz, 2017; Xia et al., 2018; Xu et al., 2019; Yang et al., 2022). Originally, SCS was mainly applied to patients with chronic pain, and later in the 1980s, Kanno et al. first reported encouraging results regarding SCS in patients with DoC (Kanno et al., 1987, 1988). The surgical procedure of SCS involves implanting electrodes along the midline of the posterior epidural space of the C2-C4 level and delivering electrical stimulation to the circuitry governing awareness (Guerra et al., 2014). The progress made by researchers has led to the preliminary exploration of the clinical factors that affect the treatment effect of SCS in DoC patients. However, the effects of different electrodes on clinical outcomes and laboratory parameters are still unclear.

Cerebrospinal fluid (CSF) is secreted by the choroid plexus of the ventricle, and its properties are similar to those of plasma and lymph, which act as diagnostic tools for many conditions affecting the central nervous system (Dhiman et al., 2019; Nakada and Kwee, 2019; Gaunitz et al., 2021; Fang et al., 2022). The main proteins in CSF are albumin and macroglobulin, and under pathological conditions such as central nervous system infection, nerve tuberculosis and nerve demyelination, the destruction of the blood–brain barrier will increase the total amount of CSF proteins (Engelhardt and Sorokin, 2009). The increase in CSF protein levels may impair CSF reabsorption, thereby affecting CSF circulation and increasing the risk of hydrocephalus (Miller et al., 1990). Recent studies have shown that DBS can induce proteomic changes in the CSF of patients, and these changes may be related to the underlying mechanism of invasive neuromodulation (Zsigmond et al., 2020). However, there are no studies on the effect of SCS on total CSF protein levels in DoC patients. We proposed that elevated CSF protein levels are associated with poor short-term outcomes, and reduced anatomic structure like sagittal diameter may contribute to it.

## Materials and methods

### Study participants

We retrospectively reviewed 66 patients with DoC who were treated with SCS at the Neurosurgery Department in Beijing Tiantan Hospital and Peking University International Hospital between December 2019 and December 2021. Considering that the patients could not understand and legally consent to treatment, the SCS procedure and the possible risks were explained to the patients’ legal representatives and/or family members. Once the realistic expectations were explained, their legal representatives and/or family members signed an informed consent document compatible with our institution’s legal and ethical committee regulations, where this trial was approved (2011-0415). When their caregivers provided written informed consent for their participation, the patients were enrolled.

### Inclusion and exclusion criteria

The inclusion criteria were as follows: (1) Clinical diagnosis of DoC (Kondziella et al., 2020; Yang et al., 2022); (2) DoC (including the VS and the MCS) lasting for more than 28 days; (3) Aged 18–70 years; (4) No significant improvement in the level of consciousness within 1 month before enrollment; and (5) Family members of the patients gave informed consent and signed the informed consent form. The exclusion criteria were as follows: (1) Coma caused by neurodegenerative diseases or postoperative coma after craniocerebral tumor operation and (2) Serious untreated complications before enrollment.

### Data collection

Data were extracted from the enrolled patients’ medical records between December 2019 and December 2021. The baseline variables included age, sex, pathogenesis, duration of DoC, diagnosis, hydrocephalus, and paroxysmal sympathetic hyperactivity (PSH). After obtaining informed consent, 2–3 ml of middle CSF samples were taken during routine lumbar puncture. The samples were kept on ice and transferred to −20°C refrigerator within 10 min.

We also collected CSF samples from patients preoperatively and on the 7th day postoperatively, and the total CSF protein level was determined by rate nephelometry. The detection process was conducted in accordance with the manufacturer’s instructions.

### Surgical procedure and stimulation protocol

**FIGURE 1:**
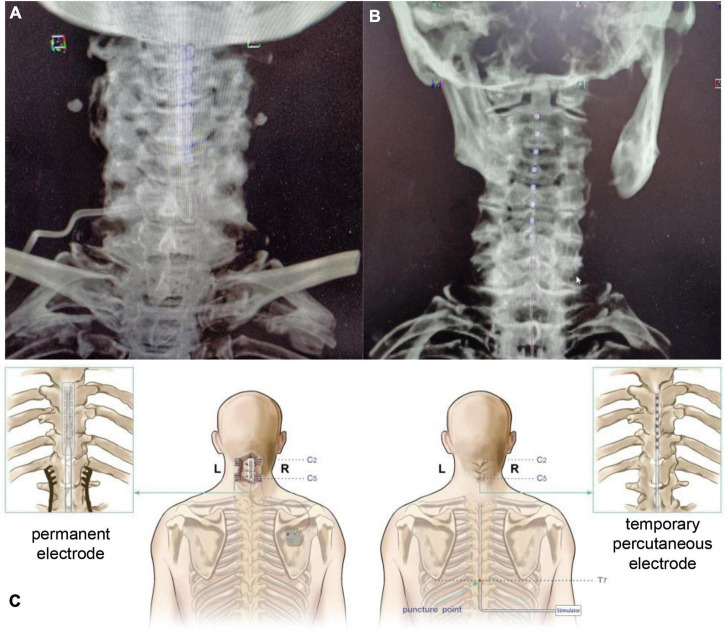
Spinal cord stimulation (SCS) surgical procedure with different electrodes. **(A)** Postoperative CT VRT reconstruction of patients receiving permanent electrode implantation. **(B)** Postoperative CT VRT reconstruction of patients receiving temporary percutaneous electrode implantation. **(C)** Schematic diagram of SCS surgeries with different electrodes. SCS, spinal cord stimulation, CT, computed tomography, VRT, volume rendering technique.

39286 electrode is 56.4 mm long, 7.6 mm wide, 1.9 mm thick, 4 mm contact length, 1.5 mm contact spacing, 42.5 mm contact tip to contact tip, and 65cm electrode tip to wire tip. In total, 3,777 electrode contact is 3 mm, the spacing is 6 mm, the length from the electrode contact tip to the contact tip is 66mm, and the total length from the electrode tip to the contact tip is 75 cm. The detailed procedure for the placement of permanent electrodes (39286, Medtronic, USA) can be found in our previous study (Yang et al., 2022; Figure 1). A midline incision at the C5 level was made under general anesthesia. After the C5 spinous process was exposed, a 2-cm-wide opening was made in the C5 spinous process and lamina. A silicone electrode model was inserted at the C2-C4 level and then replaced with a Medtronic 39286 electrode. The stimulation protocol was applied during the daytime, approximately 12 h per day. The cranial electrode was the negative pole, and the caudal electrode was the effective pole. The posterior columns were stimulated at a frequency of 70 Hz and a pulse width of 120 μs. The stimulation parameters were chosen to remain below the motor threshold, as a motor response usually occurs above 2∼2.5 V.

For temporary percutaneous electrode placement (3777, Medtronic, USA), the patient was placed in a prone position, and the T7-8 intervertebral space was positioned under the C-arm as the puncture point. The electrode (Medtronic, 3777) was inserted at the C2 level and fixed. The day after the temporary SCS operation, electric stimulation was applied to the patient’s dorsal column at a voltage of 2.5 V and a frequency of 70 Hz with a 120 μs wave width, which is consistent with the SCS treatment using permanent electrodes. The overall stimulation lasted for 21 days, and then the electrode was removed.

### Imaging data

**FIGURE 2:**
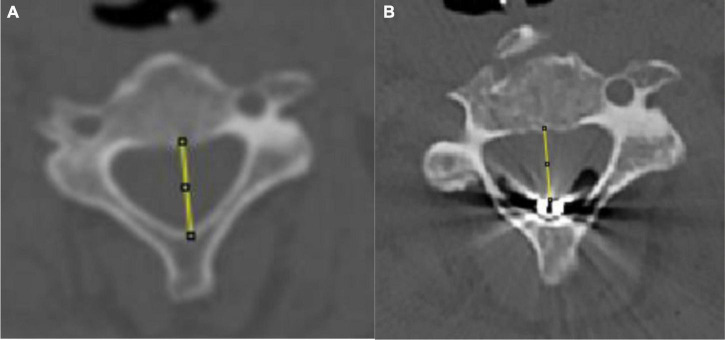
Schematic of pre- and postoperative sagittal diameter. **(A)** Preoperative sagittal diameter evaluation. **(B)** Postoperative sagittal diameter evaluation.

Before and after surgery, 64-slice spiral computed tomography (CT) (Philips, Netherlands) was used to obtain plain cervical CT scans, coronal scans, and sagittal scans of the enrolled patients. The axial images of the upper edge of the C3 vertebral body were obtained from the imaging system. The data were measured and processed independently by two senior clinicians using ImageJ (1.42) software, and the measurement results were averaged. Finally, the sagittal diameter of the spinal canal was obtained. The preoperative sagittal diameter of the spinal canal is the distance from the midpoint of the posterior border of the vertebral body to the midpoint of the posterior wall of the spinal canal at the level of the superior border of C3. The postoperative sagittal diameter of the spinal canal is the distance from the midpoint of the posterior edge of the vertebral body to the anterior edge of the stimulation electrode. Patients with a sagittal diameter reduction of more than 1.5 #39286 electrode pads (3 units) were classified as the reduced sagittal diameter group (Figure 2).

### JFK coma recovery scale–revised

The consciousness level of each patient was assessed using the CRS-R16. On the day before surgery and the 30th day after surgery, the CRS-R score was independently evaluated by 2 clinicians. For patients with an auditory score of ≤2 points, a visual score of ≤1 point, a motor score of ≤2 points, a language score of ≤2 points, a communication score of 0 points and an arousal score of ≤2 points, the diagnosis was VS. For patients with an auditory score of 3 to 4, a visual score of 2 to 5, a motor score of 3 to 5, a language score of 3, a communication score of 1 and an arousal score of ≤2, the diagnosis was MCS. Patients with improved postoperative CRS-R scores greater than 3 or who progressed to the MCS + /eMCS were classified as the improved group and otherwise regarded as poor outcome.

### Statistical analysis

SPSS 22.0 software was used to process the data. The Kolmogorov–Smirnov test was used to determine normality. Normally distributed measurement data are expressed as the mean ± standard deviation. Age was compared using an independent samples *t*-test. Chi-square tests were used for categorical variables. P for trend was tested using one-way analysis of variance (ANOVA) for continuous variables and linear-by-linear association for categorical variables. Logistic regression analysis was performed to determine the factors related to outcomes with default parameters. The significance threshold was a two-sided *P*-value less than 0.05.

## Results

### Baseline characteristics of patients with disorders of consciousness after spinal cord stimulation

Between December 2019 and December 2021, SCS was performed on 66 patients, 47 males (71.2%) and 19 females (28.8%), with an average age of 46.38 ± 13.33 years. The characteristics are summarized in Table 1. Twelve patients were allocated to the improved group, while 54 were allocated to the non-improved group. There was no significant difference in age (*P* = 0.087) or sex (0.732) between the groups. In this cohort, 27 (40.9%) cases were caused by traumatic brain injury, 11 (16.7%) cases were caused by anoxia and 28 (42.4%) cases were caused by stroke, and there was no significant difference between the groups (*P* = 0.998). We also found no significant difference in the duration of DoC (*P* = 0.839). There were significantly more patients in a MCS in the improved group (*P* = 0.003). There were significantly more patients with elevated CSF protein levels in the non-improved group (*P* = 0.022). Regarding complications, there was no significant difference in the occurrence of hydrocephalus (*P* = 0.752) or PSH (*P* = 0.752) between the groups.

**TABLE 1:** Baseline characteristics of patients with disorders of consciousness (DoC) after spinal cord stimulation (SCS).

Variables	All patients (*n* = 66)	Outcome		*P-value*
		
		Non-improved (*n* = 54)	Improved (*n* = 12)	
Age, y, mean ± SD	46.38 ± 13.33	45.06 ± 13.68	52.33 ± 10.07	0.087
Sex (%)				
Male	47 (71.2)	39 (72.2)	8 (66.7)	0.732
Female	19 (28.8)	15 (27.8)	4 (33.3)	
Pathogenesis (%)				0.998
Anoxia	11 (16.7)	9 (16.7)	2 (16.7)	
Stroke	28 (42.4)	23 (42.6)	5 (41.7)	
Trauma	27 (40.9)	22 (40.7)	5 (41.7)	
Duration (%)				0.839
3–5	39 (59.1)	31 (57.4)	8 (66.7)	
6–11	20 (30.3)	17 (31.5)	3 (25.0)	
≥12	7 (10.6)	6 (11.1)	1 (8.3)	
Diagnosis (%)				0.003*
VS/UWS	24 (36.4)	24 (44.4)	0 (0)	
MCS	42 (63.6)	30 (55.6)	12 (100)	
Elevated CSF protein, mg⋅L-1, median (IQR)	88.09 (29.55–202.64)	101.52 (34.26–227.92)	45.29 (−63.06–89.31)	0.022*
Hydrocephalus (%)	34 (51.5)	27 (50.0)	7 (58.3)	0.752
PSH (%)	32 (48.5)	27 (50.0)	5 (41.7)	0.752

DoC, disorders of consciousness; SCS, spinal cord stimulation; CSF, cerebrospinal fluid; PSH, paroxysmal sympathetic hyperactivity. *P < 0.05, significant difference.

### Clinical features of disorders of consciousness patients receiving spinal cord stimulation according to elevated cerebrospinal fluid protein levels

The clinical features of DoC patients receiving SCS according to elevated CSF protein levels are summarized in Table 2. The mean age of patients in the elevated CSF protein level group was slightly lower than that in the stable CSF protein level group, albeit not significant (*P* = 0.201). There were also no significant differences in sex (*P* = 0.099), pathogenesis (*P* = 0.532), duration of DoC (*P* = 0.379) or diagnosis (*P* = 0.062) in the elevated CSF protein level group. Complications such as hydrocephalus (*P* = 0.369) and PSH (*P* = 0.369) were also not different.

**TABLE 2:** Clinical features of patients receiving SCS according to CSF protein level variation.

Variables	Elevated CSF protein level tertiles, mg/L			P for trend
	
	T1 (*N* = 22)	T2 (*N* = 22)	T3 (*N* = 22)	
Age, y	49.05 ± 11.27	47.82 ± 11.54	42.27 ± 16.20	0.201
Sex (%)				0.099
Male	12 (54.5)	18 (81.8)	17 (77.3)	
Female	10 (45.5)	4 (18.2)	5 (22.7)	
Pathogenesis (%)				0.532
Anoxia	4 (18.2)	5 (22.7)	2 (9.1)	
Stroke	9 (40.9)	9 (40.9)	10 (45.5)	
Trauma	9 (40.9)	8 (36.4)	10 (45.5)	
Duration (%)				0.379
3–5	14 (63.6)	9 (40.9)	16 (72.7)	
6–11	5 (22.7)	10 (45.5)	5 (22.7)	
≥12	3 (13.6)	3 (13.6)	1 (4.5)	
Diagnosis (%)				0.062
VS/UWS	5 (22.7)	8 (36.4)	11 (50.0)	
MCS	17 (77.3)	14 (63.6)	11 (50.0)	
Hydrocephalus (%)	13 (59.1)	11 (50.0)	10 (45.5)	0.369
PSH (%)	9 (40.9)	11 (50.0)	12 (54.5)	0.369

SCS, spinal cord stimulation, CSF, cerebrospinal fluid, PSH, paroxysmal sympathetic hyperactivity. *P < 0.05, significant difference.

### Logistic regression analysis associated with outcomes

We analyzed the potential factors associated with outcomes in DoC patients receiving SCS treatment, and the results are shown in Table 3. Age, sex, pathogenesis and duration of DoC were all considered in the logistic regression analysis. However, after adjusting for all potential covariables, the results showed that an elevated CSF protein level was the only significant factor associated with poor outcomes (OR 1.008, 95% CI 1.001–1.016, *P* = 0.032).

**TABLE 3:** Logistic regression analysis of the risk of poor short-term outcomes after SCS in DoC patients.

Variable	Multivariate analysis		
	
	OR	95% CI	*P-value*
Elevated CSF protein	1.008	1.001–1.016	0.032*

Variables in the model that not significant: age (*P* = 0.130), sex (*P* = 0.775), pathogenesis (*P* = 0.667), duration (*P* = 0.588). SCS, spinal cord stimulation; DoC, disorders of consciousness; CSF, cerebrospinal fluid; OR, odds ratio; CI, confidence interval.

*P < 0.05, significant difference.

### The association between cerebrospinal fluid protein level and surgery-related factors

To further examine whether surgery-related factors could affect CSF protein levels, we analyzed the relationship between SCS electrodes and the sagittal diameter or CSF protein level. The results showed that in the permanent electrode group, there were significantly more patients with a reduced sagittal diameter (*P* < 0.001) and elevated CSF protein level (*P* = 0.001) than in the temporary puncture electrode group (Supplementary Table 1). We also found more DoC patients with elevated CSF protein levels in the reduced sagittal diameter group (*P* = 0.044, Supplementary Table 2).

## Discussion

In this cohort study, we found that in DoC patients receiving SCS treatment, an elevated CSF protein level was significantly associated with poor outcomes at 3 months. This result suggests that CSF protein levels may play a role in the process of rehabilitation and may be a potential biomarker in DoC patients receiving SCS treatment. Logistic regression analysis showed that elevated CSF protein levels were significantly associated with poor outcomes. We also found more DoC patients with elevated CSF protein levels among those receiving SCS treatment with permanent electrodes rather than temporary percutaneous electrodes and more DoC patients with a reduced sagittal diameter in the permanent electrodes group. Furthermore, we found that elevated CSF protein levels were significantly associated with a reduced sagittal diameter. The reason an elevated CSF protein level was more significant in the permanent electrode group was because of the larger size of the electrode pads.

In recent decades, because the pathogenesis of DoC has not been clearly stated, developments in the treatment of DoC has stagnated and different treatments have minimal effects. In recent years, with the development of neuromodulation technology, many emerging tools have been applied to the evaluation, diagnosis, and treatment of DoC (Della Pepa et al., 2013). SCS, as an invasive neuromodulation technique, is being increasingly used in the treatment of DoC (Mattogno et al., 2017; Xu et al., 2019; Wang et al., 2020; Yang et al., 2022). The underlying mechanisms of SCS for the treatment of DoC mainly include increasing cerebral blood perfusion, increasing the local glucose metabolic rate, releasing catecholamines and regulating oxidative stress (decreasing superoxide radical content) (Clavo et al., 2008; Liu et al., 2008; Wu et al., 2008; Visocchi et al., 2011; Si et al., 2018). Previous studies have shown that SCS can cause changes in CSF proteomics, and researchers have explored the key proteins that may be involved in the mechanism of SCS for treating chronic pain (Royds et al., 2020). However, the changes in total CSF protein levels after SCS treatment have rarely been studied, especially in patients with DoC.

The possible mechanisms underlying the change in total CSF protein level after SCS treatment may be due to the disruption of the blood–brain barrier by intraoperative local procedures or the disturbance of regional CSF circulation. Traditionally, CSF protects the brain and spinal cord from mechanical injury, but the understanding of the dynamic metabolism and function of CSF has rapidly improved in recent decades (Louveau et al., 2015). It has been reported that CSF is responsible for transporting hormones and waste protein *via* dynamic circulation, and increased CSF protein levels have been reported to play a role in neurodegenerative diseases such as Alzheimer’s disease, aging and other pathological processes, including subarachnoid hemorrhage and trauma (May et al., 1990; Iliff et al., 2012; Kress et al., 2014; Puvenna et al., 2016; Fesharaki-Zadeh, 2019; Coulibaly and Provencio, 2020). Disruption of the blood–brain barrier by intraoperative procedures may lead to the leakage of plasma proteins into CSF (Johnston and Teo, 2000). Previous studies have shown that nerve root compression caused by various factors can cause damage to the blood–brain barrier, therefore increasing the total CSF protein level, and the degree of increase in the total CSF protein level is positively correlated with the degree of nerve root compression (Skouen et al., 1994). SCS electrode implantation involves blunt traction on the surrounding tissue and requires removing the spinous process and part of the laminae in permanent electrode implantation procedures, which inevitably causes slight physical damage to the nerve root, as confirmed by intraoperative electrophysiological monitoring. The disruption of the blood–brain barrier caused by this physical injury may be one of the reasons for the increase in CSF protein levels in certain patients after SCS. Another reason for the increase in CSF protein levels may be due to the certain impact of SCS electrode implantation on the structure of the local CSF circulation pathway. This may be because the SCS electrode affects the normal circulation of CSF, causing a decrease in the turnover rate of CSF, which in turn leads to an increase in CSF protein levels (Chen et al., 2010). Thus, the circulation of CSF could be disrupted and cause worse outcome in those patients receiving SCS treatment. Our study also showed that after the implantation of SCS electrodes, the anatomical morphology of the implantation site is changed to a certain extent. The dura is partially compressed, the sagittal diameter of the spinal canal is reduced, and there is a significant correlation with elevated CSF protein levels (Son et al., 2018).

Furthermore, we found that elevated CSF protein levels after SCS were associated with lower patient emergence rate at 3 months. Previous studies have shown similar results: at the proteomic level, DBS, an invasive neuroregulatory procedure, can up- or downregulate the expression level of various proteins in CSF and peripheral blood and may further affect prognosis (Zsigmond et al., 2020). Additionally, Royds et al. studied the characteristics of CSF in patients with chronic pain who received SCS and found significant alterations in protein-like growth hormones. However, the sample size of that study was too small (limited to 4 patients), so the authors found no correlation between changes in CSF protein level and outcomes. Proteomic-level alterations in CSF proteins could affect the prognosis of DoC patients undergoing SCS treatment, but this requires further validation. Since elevated CSF protein levels are significantly associated with postoperative anatomical changes and affect patient outcomes, we believe that reducing the size of the electrode pads may have a potential role in improving patient outcomes. There is currently no dedicated commercial electrode developed for the treatment of DoC. Most of the current electrodes are designed for chronic pain, and larger electrode pads are selected to cover a larger area. Therefore, improving the electrodes used in surgery can further reduce anatomical changes and may further improve outcomes in patients with DoC treated with SCS.

Our study has several limitations. First, a predictive model was not generated due to the limited number of patients enrolled in this study. Second, in the current study, we tracked the change in the total CSF protein level for a short period of time and did not explore the time-course trend of the total CSF protein level, which may further clarify the effects of such factors on patient outcomes. Finally, we did not perform further proteomic analysis of CSF in this study. Future research on CSF composition may help to further elucidate this issue and explore treatment methods. Large-scale, long-term follow-up studies are still needed to validate the preliminary results obtained in this study.

## Conclusion

In this study, we found that elevated CSF protein levels were significantly associated with poor outcomes in DoC patients after SCS treatment and significantly related to a reduced sagittal diameter, which suggests that CSF protein levels may play a role in rehabilitation. The level of CSF protein may also serve as a biomarker in DoC patients receiving SCS treatment and reducing the effect of electrode pads on anatomical changes may help improve the outcomes of DoC patients receiving SCS treatment.

## Data availability statement

The raw data supporting the conclusions of this article will be made available by the authors, without undue reservation.

## Ethics statement

The studies involving human participants were reviewed and approved by the Ethics Committee of Beijing Tiantan Hospital. The patients/participants provided their written informed consent to participate in this study.

## Author contributions

QH and YY designed the study and wrote the manuscript. TL, YX, XX, YD, XC, and XG collected the data and samples. JH and JZ supervised the study. All authors contributed to the article and approved the submitted version.
