# Supplementary material for: Elevated cerebrospinal fluid protein levels associated with poor short-term outcomes after spinal cord stimulation in patients with disorders of consciousness
Source: Front Aging Neurosci. 2022 Nov 3;14:1032740. doi: 10.3389/fnagi.2022.1032740 (PMC9669419; doi:10.3389/fnagi.2022.1032740)
Supplement: Supplementary file 1 [file Table_1.DOCX]

Supplementary Material

# Supplementary Table

| Table S1. Related factors according to the SCS option. | | | |
| --- | --- | --- | --- |
| Variables | SCS option | | P value |
|  | permanent (n = 32) | puncture (n = 34) |  |
| Elevated CSF protein, mg·L-1, median (IQR) | 140.07 (56.45-285.31) | 51.84 (-5.71-101.79) | 0.001 |
| Reduced sagittal diameter (%) | 25 (78.1) | 0 (0) | <0.001 |
| Emerged Outcome (%) | 3 (9.4) | 9 (26.5) | 0.110 |
| SCS-spinal cord stimulation, CSF-cerebrospinal fluid. *P<0.05, significant difference. | | | |

| Table S2. Analysis of the sagittal diameter and elevated CSF protein level. | | | |
| --- | --- | --- | --- |
| Variable | Sagittal diameter | | P value |
|  | Reduced (n = 25) | No change (n = 41) |  |
| Elevated CSF protein, mg·L-1, median (IQR) | 67.98 (11.06-144.24) | 112.66 (54.07-234.56) | 0.044 |
| CSF-cerebrospinal fluid, *P<0.05, significant difference. | | | |
